# Molecular Characterization of a Novel Alkaline Endo-Pectate Lyase from *Paenibacillus borealis* and Over-Production in Bioreactor Realized by Constructing the Tandem Expression Cassettes in Host Genome

**DOI:** 10.3390/molecules30173612

**Published:** 2025-09-04

**Authors:** Ying Han, Xiao-Bo Peng, Shu-Ya Wei, Qi-Guo Chen, Jiang-Ke Yang

**Affiliations:** 1 Pilot Base of Food Microbial Resources Utilization of Hubei Province, College of Life Science and Technology, Wuhan Polytechnic University, Wuhan 430023, China; hanyingfyc@sina.com (Y.H.); whpupxb@sina.com (X.-B.P.); 2 College of Biological Engineering, Wuhan Technical University, Wuhan 430079, China; weishuya1011@126.com (S.-Y.W.); cqg6518@126.com (Q.-G.C.)

**Keywords:** molecular characterizing, molecular dynamics analysis, pectate lyases, tandem expression cassettes, high-density

## Abstract

Alkaline pectate lyases hold significant promise for various industrial applications, including the degumming processes in papermaking and textiles. In this study, a novel pectinase, PelA, derived from a strain of *Paenibacillus borealis*, was characterized both at the molecular level and through enzymatic analysis. This enzyme represents a distinct cluster diverging from the well-characterized *Bacillus* pectinases and exhibits molecular activity under alkaline conditions, with an optimal pH of 9.5. It can be classified as an endo-(1,4)-pectate lyase, capable of cleaving the α-1,4 glycosidic bonds of polygalacturonic acid via a trans-elimination mechanism. Notably, the addition of the metal ion Ca^2+^ did not enhance enzyme activity. To achieve high-level secretory expression and improve its economic viability for bioapplications, the gene copy number of *pelA* in the host genome was increased by constructing tandem *pelA* gene expression cassettes. Following optimization of cultivation conditions and monitoring of cell growth, the recombinant strain harboring the multi-copy *pelA* gene attained an expression level of 7520 U/mL in a bioreactor. This study successfully achieved high-level secretory expression of an alkaline pectinase, thereby enhancing its potential for industrial bioapplications and providing a reference for future research on the heterologous expression of target genes.

## 1. Introduction

Pectin is a highly abundant polysaccharide found in nature. The backbone composed of polygalacturonic acid, which can exhibit varying degrees of methyl esterification, acetylation, and other modifications. Pectin is widely distributed in plant cell walls and plays a crucial role in maintaining cellular structural stability. It significantly influences the processing of beverages, such as fruit juices, fruit wines, animal feeds, and the degumming process in papermaking [1]. Pectinase can effectively degrade the long chains of pectin, which is vital for applications in food, feed, textiles, papermaking, and natural product extraction [1,2]. Pectinase that is preferentially active in alkaline environments may serve as an ideal biocatalyst to replace chemical agents in various industrial processes that require high alkalinity for pectin removal. The incorporation of alkaline pectinase during the papermaking process can enhance pulp quality and reduce post-bleaching costs [3,4]. In comparison to traditional alkali scouring and degumming methods, biological refining offers several advantages, including environmental sustainability, fiber protection, increased refining efficiency, and reduced energy consumption. Consequently, enzyme-mediated bio-degumming has emerged as a trend in the paper industry [5,6].

Currently, alkaline pectinases have been isolated from fungi such as *Aspergillus luchuensis* [7], *Fusarium decemcellulare* [8], *Sporotrichum thermophile* [9], and some bacteria [10]. Strains from *Bacillus,* such as *Bacillus tequilensis* [11], *Bacillus subtilis* [12,13], and *Bacillus pumilus* [14], remain the primary sources of these microorganisms. However, the molecular knowledge about alkaline pectinases was also limited to Bacillus only, and *Bacillus* pectinases were well characterized. The exploration of alkaline pectinases from other bacterial sources could not only broaden our knowledge but also enrich enzyme sources for industrial application.

*Paenibacillus borealis*, from which the nominated species was originally isolated from spruce forest humus in Finland [15], represents a valuable yet underutilized resource for industrial enzymes. To enrich the pool of alkaline pectinases and enhance their heterologous expression levels in *Pichia pastoris*, thereby facilitating their industrial bioapplication, this study involved the cloning, expression, and enzymatic characterization of a putative pectinase from the *Paenibacillus borealis* strain. The strategy employed to improve gene dosage involved constructing tandem gene expression cassettes, which aimed to elevate expression levels and promote bioapplication in relevant industrial sectors.

## 2. Results

### 2.1. Comparative Phylogenetics, Structure Alignment, and Conservation Mapping of PelA Across Paenibacillus and Related Genera

**Figure 1:**
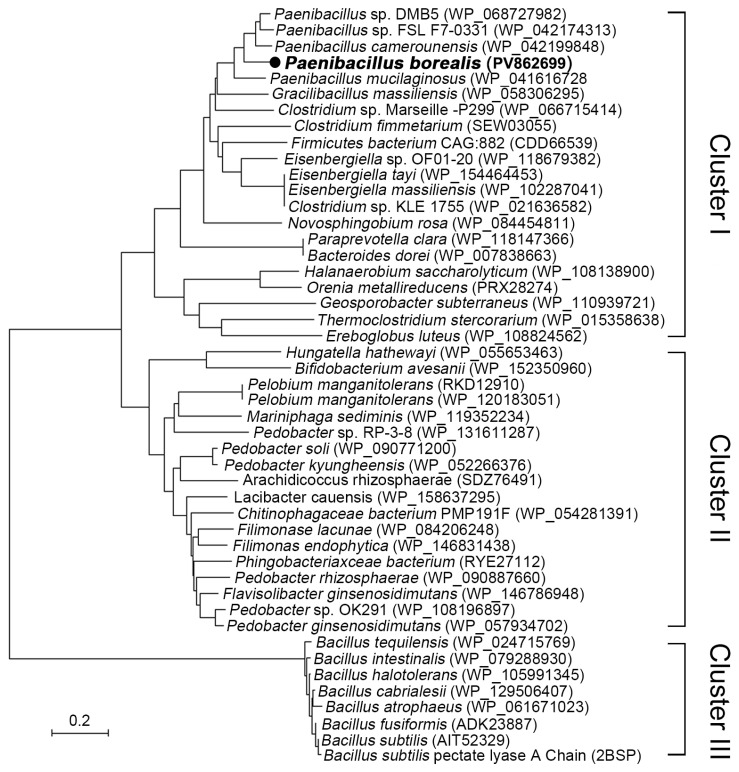
The phylogenetic analysis of pectate lyase from *Paenibacillus*, *Bacillus*, and related genera was conducted using MEGA software version 7.0. The evolutionary history was inferred through the Neighbor-Joining method, resulting in an optimal tree with a sum of branch lengths equal to 9.13. Evolutionary distances were computed utilizing the Poisson correction method.

To explore novel resources of pectate lyases, gene annotation and evolutionary analysis of pectate lyases from *Paenibacillus*, *Bacillus*, and related genera were conducted. The phylogenetic tree illustrating the relationships among the putative and identified pectate lyases from *B. subtilis* is depicted in Figure 1. As shown in Figure 1, the pectate lyases from *Paenibacillus*, *Bacillus*, and related genera are categorized into three distinct clusters. Cluster I comprises pectate lyases from *Paenibacillus*, *Gracilibacillus*, *Clostridium*, and others, all belonging to the phylum *Firmicutes*. Cluster II includes enzymes from *Pelobium*, *Pedobacter*, *Filimonas*, and others, which are classified under the phylum Bacteroidetes. With the exception of a few strains, such as *C. fimetarium* (SEW03055), *Orenia metallireducens* (PRX28274), and *Pelobium manganitolerans* (RKD12910), which were previously annotated as polygalacturonases, most proteins in these two clusters remain uncharacterized, suggesting that strains in these clusters may harbor rich enzyme resources. Cluster III consists of strains from the genus *Bacillus*, which diverges phylogenetically from Clusters I and II. This cluster includes the three-dimensional structure-resolved pectate lyase (PDB: 2BSP) from *B. subtilis*.

**Figure 2:**
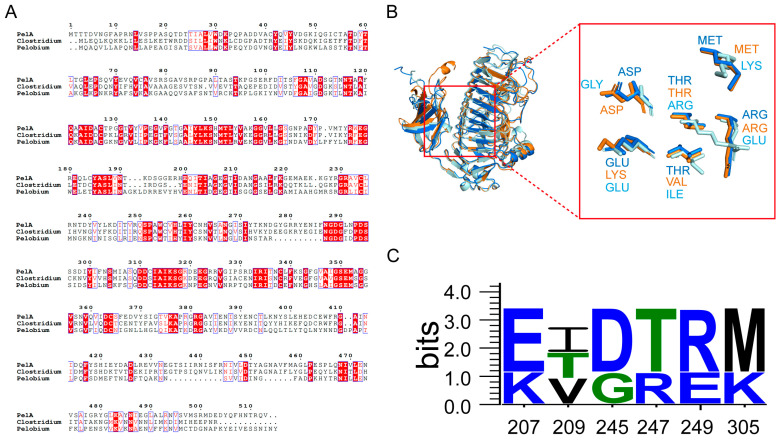
The sequence and structural alignment of pectinases is presented as follows: (**A**) The conservation analysis of PelA (*Paenibacillus*), SEW03055 (*Clostridium*), and RKD12910 (*Pelobium*) reveals that the red background indicates fully conserved amino acids, while the blue boxes represent specific structural or functional regions. (**B**) The structural alignment of pectinases PelA, SEW03055, and RKD12910 is illustrated, with PelA, SEW03055, and RKD12910 represented in blue, orange, and pale cyan, respectively. Specific amino acid residues involved in the active site or ligand interactions are highlighted. (**C**) The conservation analysis of key residues is also included.

The structural alignment and conservation analysis of amino acid sequences for PelA and three other pectinases—*C. fimetarium* SEW03055 (Cluster I); *P. manganitolerans* RKD12910 (Cluster II); and *B. subtilis* 2BSP (Cluster III)—revealed distinct patterns of residue conservation (Figure 2). The results of the multiple sequence alignment indicate that the enzymes PelA, SEW03055, and RKD12910 exhibit a significant degree of conservation in their amino acid sequences. However, notable differences are observed in certain regions of the enzymes PelA, SEW03055, and RKD12910 from Clusters I and II when compared to 2BSP (Cluster III), which may reflect evolutionary or functional divergence among these enzymes. The consistency observed in positions 310–350, which are implicated in the enzyme’s active site or substrate binding, contrasts with the variability noted between positions 420 and 460, potentially linked to species-specific functions (Figure 2A).

*P. borealis* PelA exhibits structural homology with enzymes from the PL family 1 (Figure 2B). A notable structural characteristic of PelA is the predominance of a right-handed parallel α-helix, which is formed by three parallel β-sheets. These three β-sheets are interconnected by loops or turns, resulting in a deep barrel-shaped structure that creates a catalytic cleft adjacent to the domain. The active and substrate-binding amino acids are situated within a lengthy loop extending from the core structure. In the superimposed structures of the clefts, the three alkaline amino acids K220, R231, and R249 of PelA are positioned as catalytic residues (Figure 2B). The adjacent amino acids serve as conservatively located substrate-binding sites along the clefts. Among these, alkaline amino acids such as K207 (LYS), R247 (ARG), R249 (ARG), and K305 (LYS) are present in higher proportions (Figure 2C).

### 2.2. Definition of Enzymatic Activity Classifies PelA as an Alkaline Endo-Pectate Lyase

**Figure 3:**
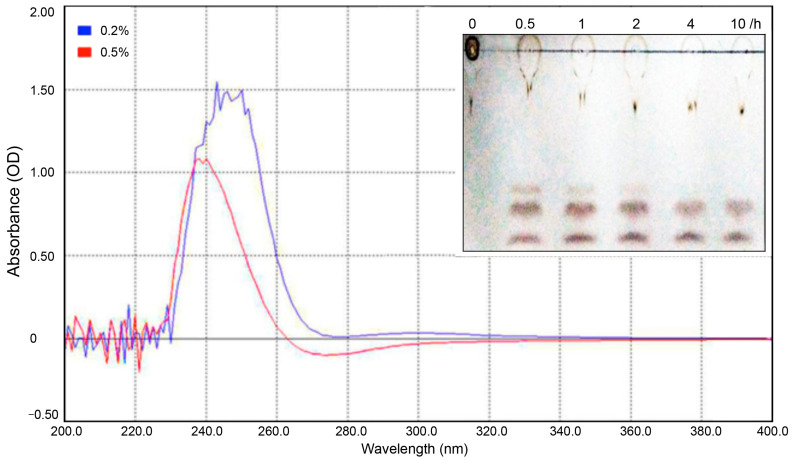
This study analyzes the products of PGA digested with the pectinase PelA. The digested products of PGA were scanned at wavelengths ranging from 200 nm to 400 nm. Additionally, a TLC analysis of the PGA products is presented. Line 1 represents the PGA substrate, while lines 2 to 6 correspond to the PGA products digested by PelA at 55 °C for durations of 0.5 h, 1 h, 2 h, 4 h, and 10 h, respectively.

**Figure 4:**
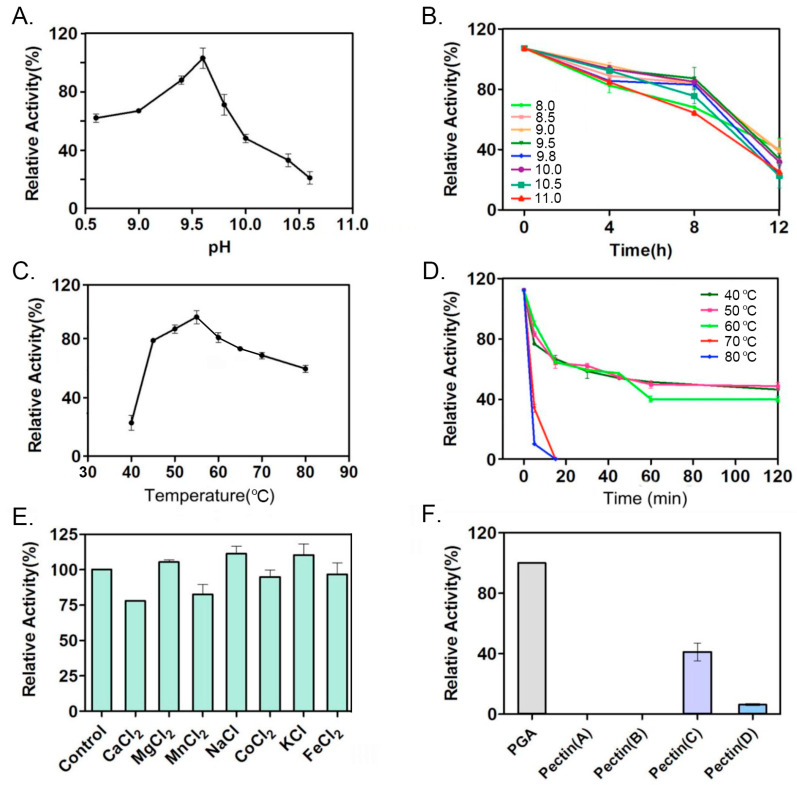
The enzymatic characteristics of pectinase PelA were evaluated in several aspects. (**A**) The optimal pH for PelA activity was determined. (**B**) The stability of PelA was assessed by incubating it in buffers with varying pH levels, and the remaining activity was measured at specific intervals. (**C**) The optimal temperature for PelA activity was identified. (**D**) The stability of PelA was also analyzed by incubating it at different temperatures, with remaining activity checked at regular intervals. (**E**) The activity of PelA was examined by incubating the enzyme with various metal ions. (**F**) Finally, the activity of PelA on different pectin substrates was investigated.

The putative pectate lyase gene pelA from *P. borealis* was successfully cloned and expressed. The enzymatic characteristics of PelA were thoroughly evaluated in this study (Figure 3 and Figure 4). To determine the type of pectate lyase, polygalacturonic acid (PGA) was employed as the substrate. The resulting digested products were analyzed using ultraviolet-visible spectrophotometry in the wavelength range of 200–400 nm, followed by thin-layer chromatography (TLC) analysis. As previously reported [16], pectate lyase cleaves the α-1,4 glycosidic bonds of polygalacturonic acid through a trans-elimination mechanism, resulting in the formation of C4:C5 unsaturated products. This double bond exhibits an absorbance peak within the 230–270 nm wavelength range. As illustrated in Figure 3, the digested products of PGA (at concentrations of 0.2% and 0.5%) demonstrate a strong ultraviolet absorption peak at 240–270 nm, corroborating the aforementioned hypothesis and indicating that the enzyme PelA from *P. borealis* can be classified into pectate lyase clusters. The TLC analysis revealed that the digested products of PGA consist of three components, corresponding to mono-, di-, and tri-saccharides, respectively. This finding indicates that the enzyme PelA can endo-digest polygalacturonan to yield oligogalacturonic products. Therefore, the enzyme PelA can be classified as an endo-pectate lyase.

This study investigates the enzymatic characteristics of PelA. As illustrated in Figure 4A,B, PelA from *Pseudomonas borealis* is identified as a typical alkaline pectinase, exhibiting an optimal pH of 9.5. When incubated in an alkaline buffer above pH 9.0 for up to 4 h, PelA retained 80% of its activity. The optimal temperature for PelA activity was determined to be 55 °C (Figure 4C), and the enzyme demonstrated notable thermal stability (Figure 4D). The presence of metal ions such as Mg^2+^, Na^+^, and K^+^ enhanced enzyme activity by 5%, 11%, and 9%, respectively, while Ca^2+^, Mn^2+^, and Co^2+^ reduced activity by 23%, 18%, and 6%, respectively (Figure 4E). Furthermore, PelA exhibited a preference for pectin substrates with varying degrees of esterification (Figure 4F), showing the highest activity on polygalacturonic acid (PGA). Among naturally occurring pectins with different degrees of esterification, PelA favored medium-esterified pectin (66–69%).

### 2.3. Molecular Docking and Constant-pH Molecular Dynamics Simulations

**Figure 5:**
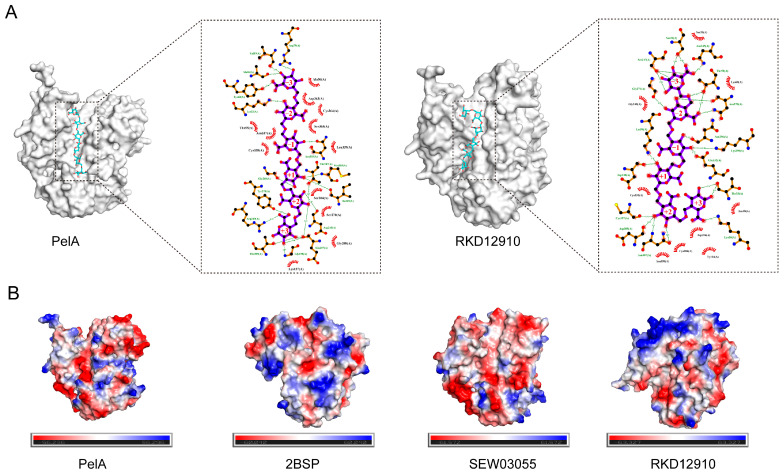
The molecular docking of pectinase with the substrate hexagalacturonic acid and the electrostatic potential maps of four distinct pectinase structures are presented. (**A**) This section illustrates the molecular docking of pectinases PelA and RKD12910 with hexagalacturonic acid at subsites −3, −2, −1, +1, +2, and +3, respectively. (**B**) The electrostatic diagrams for pectinases PelA, 2BSP, SEW03055, and RKD12910 are depicted, highlighting the regions of positive (blue), negative (red), and neutral (green/white) charges.

Molecular docking studies of PelA with hexagalacturonic acid demonstrated that PelA binds to the substrate at subsites −3, −2, −1, +1, +2, and +3, establishing hydrogen bond interactions at each position, with a binding energy of −8.5 kcal/mol. Similarly, RKD12910 forms hydrogen bonds with the six subsites of hexagalacturonic acid, exhibiting a binding energy of −8.9 kcal/mol (Figure 5A). The electrostatic potential maps of the four distinct pectinase structures (PelA, 2BSP, SEW03055, and RKD12910) (Figure 5B) provide valuable insights into the charge distribution across their surfaces, which is essential for understanding substrate binding and catalytic activity. These maps reveal distinct patterns of positive (blue) and negative (red) charge regions, with neutral areas (green/white) varying among the structures. The enzymes PelA, SEW03055, and RKD12910 exhibit a tendency towards neutrality, whereas 2BSP displays a significantly positive charge in its active cleft. Such differences likely reflect variations in amino acid composition and protonation states, influenced by the local environment or pH conditions. Regions with concentrated negative charges may indicate potential binding sites for positively charged substrates or cofactors, while clusters of positive charge could facilitate interactions with negatively charged pectin components. The heterogeneity in electrostatic potential among the four pectinases suggests structural adaptations that may correlate with their specific catalytic efficiencies or substrate preferences.

**Figure 6:**
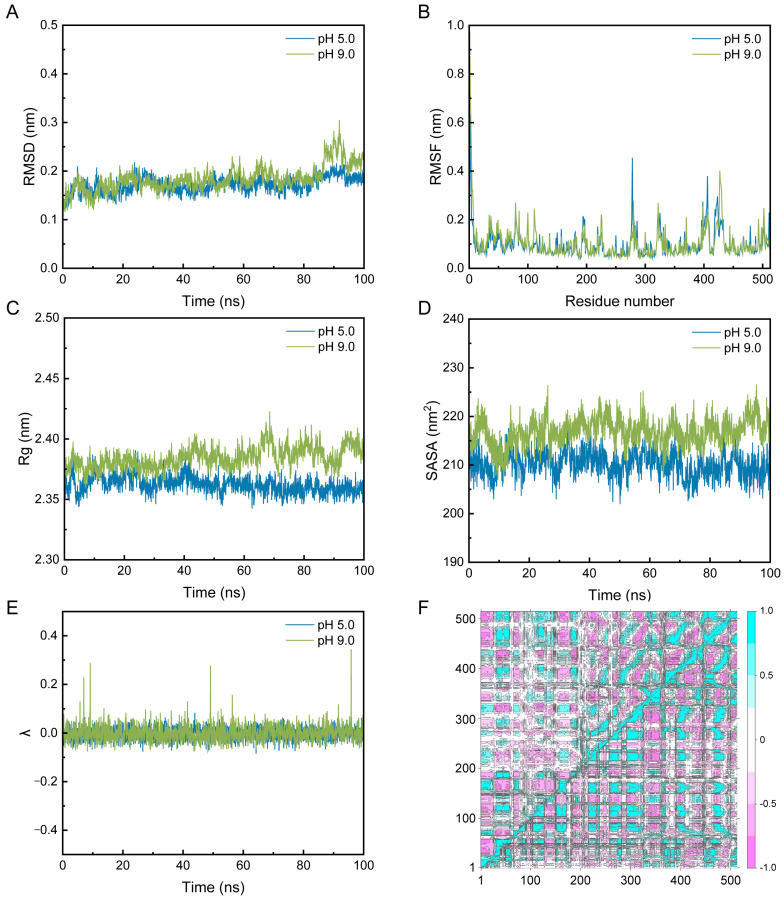
The constant-pH molecular dynamics simulations of PelA were conducted to analyze its structural properties. The results include the Root Mean Square Deviation (RMSD) (**A**), Root Mean Square Fluctuation (RMSF) (**B**), Radius of Gyration (Rg) (**C**), Solvent Accessible Surface Area (SASA) (**D**), λ (**E**), and Dynamic Cross-Correlation Map (DCCM) (**F**) of PelA at pH 5.0 and 9.0, respectively. In the DCCM analysis, the upper left quadrant displays the DCCM of PelA at pH 5.0, while the lower right quadrant corresponds to pH 9.0. Figure (**E**) illustrates that λ fluctuates between 0 and 0.4 for PelA at pH 5.0 (blue) and pH 9.0 (green). Values approaching 0 indicate a predominantly deprotonated state, whereas values near 1 signify a protonated state. The DCCM map effectively highlights the differences in inter-residue contacts under the two pH conditions.

The constant-pH molecular dynamics analysis of PelA was performed at pH values of 5.0 and 9.0. The root-mean-square deviation (RMSD) plots indicate that the systems reached equilibrium after approximately 20 ns, with RMSD values stabilizing between 0.2 and 0.4 nm (Figure 6A). Notably, an increase in RMSD was observed at pH 9.0 compared to pH 5.0, suggesting greater conformational flexibility at higher pH levels, likely due to altered protonation states of the titratable residues in alkaline pectinase PelA. Additionally, root-mean-square fluctuation (RMSF) analysis showed that residue flexibility varied across the protein sequences, particularly at positions 270–300 and 390–440, which correspond to the activity cleft and the region associated with conformational stability. Peaks indicated higher mobility at pH 9 (Figure 6B). These findings suggest that alkaline conditions may enhance local structural dynamics. The radius of gyration (Rg) and solvent-accessible surface area (SASA) plots at pH 5 and 9 clearly demonstrate that the structure of PelA is more pronounced, with increased solvent exposure at pH 9.0, consistent with the observed flexibility throughout the structure (Figure 6C,D). The λ plot illustrates the deprotonated (0) or protonated state (1) under pH 5.0 and pH 9.0 conditions (Figure 6E). The observed range suggests a mixture of protonation states. At pH 5.0, the wavelength (λ) remains relatively stable with minor fluctuations, indicating a consistent protonation environment. In contrast, at pH 9.0, the λ values exhibit more pronounced peaks, suggesting increased transitions between protonation states, likely due to the higher pH favoring the deprotonation of acidic residues. This pH-dependent behavior reflects the protein’s dynamic response to environmental changes, which may influence its structure and function. The dynamic cross-correlation matrices (DCCM) map highlights differences in inter-residue contacts between the two pH conditions, with notable changes in the 200–400 residue region, underscoring pH-induced structural rearrangements (Figure 6F).

### 2.4. Improving the Expression Level of PelA Through Tandem Expression Cassettes Construction

**Figure 7:**
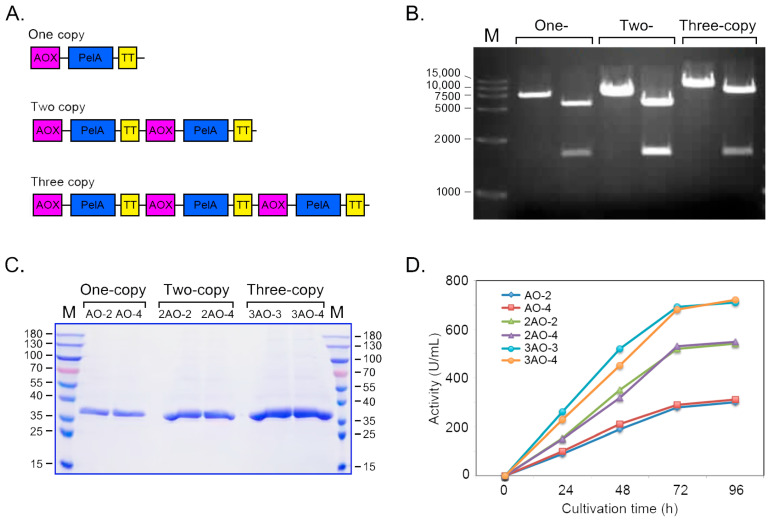
Expression of pectinase PelA was achieved by constructing tandem expression cassettes. (**A**) A schematic diagram illustrating the organization of the PelA gene expression cassettes is presented. (**B**) The sizes of the one-, two-, and three-copy concatemers of the expression cassettes were assessed. (**C**) SDS-PAGE analysis was conducted to evaluate the proteins expressed in the culture of BtChy recombinants. (**D**) The enzyme activity of the recombinants containing different copies of the pelA gene in their genomes was measured.

To enhance the gene dosage of *pelA* in the host genome, a series of recombinant plasmids carrying tandem *pelA* gene expression cassettes—namely; pAO-pelA; pAO-Du-pelA; and pAO-Tri-pelA—were constructed (Figure 7A,B). *Pichia* recombinants with varying pelA gene copy numbers in their genomes were obtained and quantitatively analyzed using quantitative PCR (QPCR) (Table 1). As indicated in Table 1, the recombinants AO-2 and AO-4 possess one copy of *pelA* in their genomes. The strains 2AO-2 and 2AO-4 contain two copies, while the strains 3AO-3 and 3AO-4 harbor three copies of *pelA* in their genomes, respectively. The expression levels of *pelA* in these Pichia recombinants were evaluated in this study. As shown in Figure 7C,D, the expression levels gradually increased with the elongation of culture time, peaking at 96 h. The strains with one copy of *pelA* exhibited an enzyme activity of 280 U/mL of culture. The expression levels of the two-copy and three-copy recombinants were significantly higher than those of the one-copy strains. Notably, the three-copy recombinants generally exhibited the highest enzyme activity at the 96 h time point, reaching an activity level of 720 U/mL of culture in the flask.

### 2.5. Expression of PelA in Bioreactor

**Figure 8:**
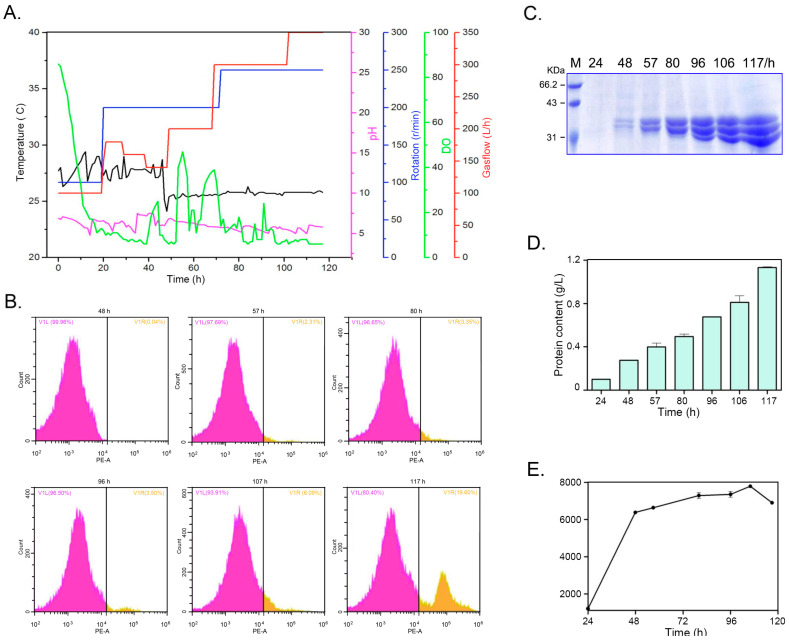
The cultivation of yeast recombinants and the expression of PelA in a 14 L bioreactor were investigated. (**A**) The parameters varied throughout the entire culture period. (**B**) Cell counts were obtained using a flow cytometer during the cultivation in the bioreactor. The V1L plot represents the proportion of living cells, while the V1R plot indicates the proportion of dead cells. (**C**,**D**) illustrate the protein profiles and content in the supernatant of the culture within the bioreactor. (**E**) The pectinase activity of the culture in the bioreactor was measured. Samples were aliquoted at intervals of 24 h, 48 h, 57 h, 80 h, 96 h, 106 h, and 117 h.

To evaluate the potential for large-scale production of PelA, *Pichia* recombinants harboring three copies of the *pelA* gene were cultivated in a bioreactor. The cultivation parameters—including dissolved oxygen (DO); gas flow; rotation speed; temperature (T_m_); and pH of the medium—were meticulously controlled; as illustrated in Figure 8A. During the initial 40 h, Tm was maintained at approximately 28 °C, after which it stabilized around 25 °C. The pH of the medium was consistently regulated at approximately 5.5 throughout the entire cultivation period. The DO was adjusted through a cascaded control of agitation rate, airflow, and methanol feeding. Cell density was monitored using a flow cytometer during cultivation in the bioreactor (Figure 8B). The data indicate that, in the early stages, nearly all cells were active. However, as cultivation time extended, dead cells became detectable, particularly at 117 h. Improved control over the cultivation parameters resulted in the expression of PelA in the bioreactor significantly exceeding that observed in flask cultures. As demonstrated in Figure 8C,D, the protein content gradually increased to 1.08 g/L by 117 h, with enzyme activity peaking at 7520 U/mL at the 106 h time point (Figure 8E). This activity was nearly ten-fold greater than that observed in flasks (see Figure 7). However, a decline in activity was noted at 117 h.

## 3. Discussions

### 3.1. The Alkaline Pectinase PelA from P. borealis Represents a Cluster Divergent from Bacillus Pectinases

Alkaline pectate lyase serves as an environmentally friendly biocatalyst in the degumming and papermaking processes [6,14]. Although alkaline pectate lyases have been isolated from fungi and various bacteria [7,10,17,18], the primary sources continue to be species within the *Bacillus* genus, including *B. tequilensis* [11], *B. subtilis* [12,13,19], *B. pumilus* [14], and *B. amyloliquefaciens* [20]. The identification of alkaline pectinase genes in other bacterial species remains limited, with only two pectinases reported from *Paenibacillus polymyxa* [21,22]. Phylogenetic analysis reveals that strains from *Paenibacillus* and related genera harbor a wealth of pectinase resources, which can be categorized into three clusters: I, II, and III (Figure 1). Except for a few strains, such as *Clostridium fimetarium* (SEW03055), *Orenia metallireducens* (PRX28274), and *Pelobium manganitolerans* (RKD12910) from Clusters I and II that have been previously annotated, most proteins in these clusters remain uncharacterized, indicating that strains within these clusters may possess abundant enzymatic resources.

Enzymatic characterization demonstrated that PelA can effectively digest polygalacturonic acid into mono-, di-, and tri-saccharides. As previously reported [21], pectate lyase can break polygalacturonic acid through a trans-elimination mechanism, resulting in C4:C5-unsaturated products that exhibit an absorbance peak within the 230–270 nm wavelength range. As illustrated in Figure 3, the digested products of PGA display a strong ultraviolet absorption peak at 240–270 nm, which corroborates the aforementioned speculation and indicates that the enzyme PelA from *P. borealis* is a type of endo-pectate lyase. Further characterization revealed that PelA has an optimal pH of 9.6 (Figure 4). Interestingly, the metal ion Ca^2+^ did not stimulate an increase in PelA activity, contrary to findings reported for other types of pectinases [23]. This observation suggests that the pectinase can exert its potential for enzymatic ramie degumming under alkaline conditions without the need for additional Ca^2+^, a phenomenon also reported for the alkaline pectate lyase, BspPelA [24].

### 3.2. Molecular Characterization Reveals Alkaline Adaptation and Structural Divergence in PelA Clusters from Bacillus Pectinases

Pectinases exhibit a characteristic parallel α-helix topology, wherein the β-strands are arranged into a prominent right-handed coil [23,25]. This study reveals that the principal differences among PelA and related structures are attributed to the size and conformation of the loops that extend from and envelop the parallel α-helix core. Sequence alignment demonstrates that, within the superimposed structures of the cleft region, amino acids such as K207 (LYS), R247 (ARG), R249 (ARG), and K305 (LYS) serve as conservatively positioned sites for substrate binding along the clefts (Figure 2). The charge distribution, as indicated by the electrostatic potential maps, reveals that the cleft and adjacent regions of PelA, SEW03055, and RKD12910 tend to exhibit neutrality, whereas *Bacillus* pectinase 2BSP functions optimally in alkaline environments. The heterogeneity in electrostatic potential among the four pectinases suggests structural adaptations that may correlate with their specific catalytic efficiencies or substrate preferences. Furthermore, molecular dynamics analysis indicates that most isozymes demonstrate catalytic activity under alkaline conditions, with an optimal pH of approximately 9.0 (Figure 6).

In this study, the addition of Ca^2+^ to the reaction mixture did not enhance PelA activity, which contrasts with findings related to other types of pectate lyases [23]. A similar phenomenon was observed with pectinase BspPelA, where the activity measurements did not require the addition of calcium [19]. Structural analysis of PelA and related enzymes reveals that some highly conserved Ca^2+^ binding residues and secondary structures are altered in PelA, making it difficult for the enzyme to coordinate with Ca^2+^ as seen in other pectate lyases. Molecular docking studies indicate that the interaction between the active cleft of PelA and hexagalacturonic acid involves direct enzyme-substrate interactions, rather than relying on Ca^2+^ ions for bridging, particularly in the extremely alkaline environment shared by PelA and RKD12910 (Figure 5A).

### 3.3. Improving the Gene Dosage of PelA and High-Density Cultivation Realized Its High-Level Production

To realize industrial applications, achieving a high-level expression is a prerequisite for enhancing economic competitiveness. In this study, a method to improve the heterologous expression level of the *pelA* gene in *Pichia pastoris* by increasing the gene dosage within the host’s genome was employed. The gene copy number is a critical factor that correlates with gene expression [26]. Previous studies have demonstrated that increasing the copy number of a gene in the host genome can correspondingly elevate its expression level [27]. In this study, the tandem expression cassettes for the *pelA* gene were constructed, and successful *Pichia* recombinants containing one, two, or three copies of *pelA* in their genomes were obtained. The strains with three copies of *pelA* exhibited nearly three-fold activity compared to those with one copy (Figure 7), further indicating that enhancing gene dosage can effectively improve gene expression levels. Following optimization of parameters, the strains carrying three copies of *pelA* in their genomes achieved an expression level of 7520 U/mL in the bioreactor (Figure 8). which is significantly higher than the primary expression levels observed for the pectate lyase gene in *Bacillus* strains [24,28].

The high-density cultivation was conducted in a 14 L bioreactor with the functional volume of 7 L (Eppendorf). In the glycerol culture phase, the parameters were maintained at a temperature of 28 °C, and a pH of 5.5, and the dissolved oxygen (DO) was kept higher than 15%. During the methanol-induced expression phase, the temperature was adjusted to 25 °C, methanol was flowed into the broth at a rate of 2 mL/h·L^−1^, and the DO level exceeded 15% by increasing the rotation and gas flow (Figure 8A). In the cultivation process, at the 117 h time point, although the total protein content in the culture was higher than that at 106 h, the enzyme activity was lower (Figure 8). Consequently, cell viability throughout the entire culture process was assessed using a flow cytometer. As illustrated in Figure 8B, at 57 h, the density of living cells gradually increased to a high value, while dead cells constituted only about 2.31% of the total cell population. With the extension of the cultivation time, the proportion of dead cells gradually increased to 19.6% at 117 h, approximately three times that observed at the 106 h time point. It is speculated that the decline in cell viability during the fed-batch process may be attributed to either nutrient deficiency or the accumulation of toxic metabolic by-products, which is the primary reason for the decrease in enzyme activity. Future work should focus on enhancing cell viability in the bioreactor as an effective strategy to improve the expression of target proteins.

## 4. Materials and Methods

### 4.1. Phylogeny Analysis, Structural Alignment, and Conservation Analysis of Pectate Lyases

The evolutionary analysis of 47 pectate lyases from *Paenibacillus*, *Bacillus*, and related genera were collected from GenBank with the accession number bracketed in Figure 1. The evolutionary history was inferred using the neighbor-joining method calculated with 100 replicates using MEGA7 software [29], and evolutionary distances were computed using the Poisson correction method. The phylogenetic tree was constructed based on these evolutionary distances using the tree program. The branch length indicates the evolutionary distance marked in Figure 1.

The three-dimensional structures of *P. borealis* PelA, *Clostridium fimetarium* (SEW03055), and *Pelobium manganitolerans* (RKD12910) were obtained through modeling using AlphaFold3 “https://alphafold.com (accessed on 29 March 2025)”. The average pLDDT scores of *P. borealis* PelA, *C. fimetarium* SEW03055, and *P. manganitolerans* RKD12910 were 92.3%, 91.7%, and 90.5%, respectively. The pAE plots confirmed low positional uncertainty in functional domains. PROCHECK “https://www.ebi.ac.uk/thornton-srv/software/PROCHECK/ (accessed on 30 March 2025)” showed >97% residues in favored Ramachandran regions. To align the structures of PelA with those of three other pectinases, namely *B. subtilis* (2BSP), *C. fimetarium* (SEW03055), and *P. manganitolerans* (RKD12910), PyMOL was used for visualization, and conservation analysis was conducted using ESPript 3.0 “https://espript.ibcp.fr (accessed on 30 March 2025)” and WebLogo 3 “https://weblogo.threeplusone.com (accessed on 30 March 2025)”. The structural analysis was performed using PyMOL software “https://pymol.org (accessed on 30 March 2025)”.

### 4.2. Gene Cloning, Expression, and Enzymatic Characterization of PelA

The putative pectate lyase gene *pelA* from the genome of *Pseudomonas borealis* was cloned, and the sequence has been deposited in GenBank with ID: PV862699. Subsequently, it was subcloned into the *Pichia pastoris* expression vector pAO815 to generate the recombinant plasmid pAO-pelA, which was then transformed into the GS115 strain via electroporation. The PelA recombinant colonies were cultured and induced for expression for approximately 96 h in *Pichia pastoris*, as described by Mattanovich et al. [30]. The culture supernatant was collected by centrifugation and subsequently purified using a Superdex 75 column (10 × 300 mm) on an ÄKTA pure machine (GE Healthcare, Rydalmere, NSW, Australia). About 2.0 mL of protein solution (Tris-HCl 50 mmol/L, NaCl 100 mmol/L, PMSF 1 mmol/L, pH 8.0) was loaded into the column, and flow-through with elution buffer (Tris-HCl 50 mmol/L, NaCl 200 mmol/L, PMSF 1 mmol/L, pH 8.0) with the flow rate of 0.5 mL per min. The fraction with the strongest absorbance peak was collected and then dialyzed for the following experiments.

To measure the activity of the PelA enzyme, the reaction mixture consisted of 900 μL of 0.33% (*w*/*v*) polygalacturonic acid (PGA) dissolved in Gly-NaOH buffer (pH 9.0) and 100 μL of diluted enzyme, followed by incubation at 50 °C for 10 min. The reaction was terminated by boiling the mixture in water for 5 min. A control reaction mixture containing 100 μL of heat-inactivated enzyme was also prepared. To determine the optimal reaction temperature for *PelA*, a series of temperatures (40 °C, 45 °C, 50 °C, 55 °C, 60 °C, 65 °C, 70 °C, and 80 °C) were set, about 100 μg of protein per reaction was aliquoted, and the activity of *PelA* at these temperatures was measured. To assess the thermal stability of the enzyme, *PelA* was incubated at 40 °C, 50 °C, 60 °C, 70 °C, and 80 °C for various durations (10 min, 30 min, 60 min, 90 min, 120 min, and 240 min), after which the remaining activity was measured. To investigate the effect of pH on *PelA* activity, a series of reaction buffer pH values (8.0, 8.5, 9.0, 9.5, 9.8, 10.0, 10.5, and 11.0) were established. Correspondingly, to assess the pH stability of the enzyme, *PelA* was incubated in the aforementioned buffers for 4, 8, and 12 h, after which the remaining activity was measured. To determine the influence of metal ions on enzyme activity, the salts MgCl_2_, FeCl_2_, MnCl_2_, CaCl_2_, CuCl_2_, and ZnCl_2_ were dissolved in Gly-NaOH buffer (pH 9.6) to a final concentration of 2 mmol/L. For every factor, three samples were collected and measured. The average values were plotted in the figures. Additionally, PGA and natural pectin with varying degrees of esterification were dissolved in Gly-NaOH buffer (pH 9.6) and subsequently used as substrates to investigate the specificity of PelA.

Pectate lyase activity was evaluated by measuring the reducing sugars released from polygalacturonic acid (PGA), which served as the substrate. One unit of enzyme activity is defined as the amount of enzyme that releases 1 μmol of reducing sugar per minute under specified assay conditions. Enzyme activity was quantified using the DNS method, and the products released from PGA by the enzymes were analyzed through thin-layer chromatography. The developing solvent was a mixture of n-butanol, water, and acetic acid in a volume ratio of 5:3:2. The developer was prepared by mixing concentrated sulfuric acid with ethanol in a volume ratio of 1:19. For each sample, the activity was analyzed in triplicate. The average value and the standard deviation (SD) among these three replicates were calculated, and the SD value was illustrated as an error bar.

### 4.3. Construction of the Tandem Expression Cassettes of PelA

The *pelA* gene was inserted into the *Pichia pastoris* expression vector pAO815 to generate the plasmid pAO-PelA. Subsequently, the expression cassette 5′AOX_1_-PelA-TT was excised from the recombinant plasmid pAO-PelA through *Bgl* II and *BamH* I digestion and ligated into the vector pAO-PelA, resulting in the plasmid pAO-Du-PelA, which contains two copies of the expression cassette 5′AOX_1_-PelA-TT. Additionally, the three-copy expression cassette plasmid pAO815-Tri-PelA was constructed by inserting the expression cassette 5′AOX_1_-PelA-TT into the plasmid pAO815-Du-PelA. These recombinant plasmids were subsequently transformed into *Pichia pastoris* via electroporation using a Gene Pulser (Bio-Rad, Richmond, CA, USA) in accordance with the manufacturer’s instructions.

### 4.4. Detection of Multiple Copies of the Integration by Quantitative PCR

The real-time quantitative PCR (QPCR) method was used to quantify the copy number of the *PelA* gene in the genome of *P. pastoris*. The primers PelF2 (5′-GGACAGGTGCACGTCTACAA-3′) and PelR2 (5′-CCGTTCAACAAGGTACCGGA-3′) were designed according to the sequence of pel AO. The primers gapdF (5′-TTGTCGGTGTCAACGAGGAG-3′) and gapdR (5′-GGTCTTTTGAGTGGCGGTC-3′) were designed based on the sequence of the glyceraldehyde triphosphate dehydrogenase gene (*gapd*) (GeneBank Accession No. U62648). The process for QPCR was conducted mainly according to the description by Lee et al. and revised by Abad et al. [31,32].

### 4.5. Inducible Expression of PelA in Bioreactor and Yeast Cell Viability Counting

The inducible expression of PelA in the bioreactor was primarily conducted following the protocols established by Mattanovich et al. [30] with subsequent modifications by Yang et al. [33]. Throughout the cultivation process, fermentation parameters were maintained at a temperature of 28 °C, a pH of 5.5, and a dissolved oxygen level exceeding 15%. During the methanol-induced expression phase, the temperature was adjusted to 25 °C, and methanol was flowed into the broth at a rate of 2 mL/h·L^−1^. Cell viability in the bioreactor was assessed using flow cytometry with CytExpert (Beckman, Brea, CA, USA). Dead cells were stained with propidium iodide (PI), which emitted strong red fluorescence at a wavelength of 660 nm, while living cells remained unstained and emitted only weak autofluorescence. The separation of living and dead cells was achieved by comparing differences in fluorescence intensity.

### 4.6. Molecular Docking and Constant-pH Molecular Dynamics Analysis

Molecular docking was conducted using the AutoDock Vina software “https://vina.scripps.edu (accessed on 31 March 2025)”. Prior to docking, both the small molecule substrates and enzymes underwent preprocessing. The docking center was established at coordinates x = 0.8, y = 4.4, and z = −1.9, with a cubic box size of 80 Å edge length and a spacing step of 0.375 Å. Conformations were ranked based on their docking scores, and the optimal conformation was chosen for binding mode analysis.

Constant pH molecular dynamics (CpHMD) simulations were conducted to investigate the pH-dependent behavior of the system. All simulations utilized the GROMACS 2023.4 software package with the CHARMM36 force field “https://www.gromacs.org (accessed on 31 March 2025)”. The protonation states of titratable residues were sampled using a hybrid Monte Carlo/molecular dynamics approach as implemented in the CpHMD module. The initial protein structure of PelA was obtained from AlphaFold3. The system was solvated in a cubic box containing TIP3P water molecules. Sodium and chloride ions were added to neutralize the system and achieve a physiological ionic strength of 0.15 mol/L. The simulation pH was maintained at 5.0 and 9.0 using the λ-dynamics method, with protonation state transitions attempted every 1000 steps. The system was energy-minimized using the steepest descent algorithm until convergence was achieved (less than 1000 kJ/mol/nm). Equilibration was performed in two stages: (1) A 100 ps NVT simulation at 300 K utilizing the Berendsen thermostat, and (2) a 100 ps NPT simulation at 1 bar employing the Parrinello-Rahman barostat. Production runs were conducted for 100 ns within the NPT ensemble, with a time step of 2 fs. Long-range electrostatic interactions were addressed using the Particle Mesh Ewald (PME) method, with a cutoff of 1.0 nm for both electrostatic and van der Waals interactions. All bonds involving hydrogen atoms were constrained using the LINCS algorithm.

## 5. Conclusions

The molecular characterization of a novel alkaline endo-pectate lyase from *Pseudomonas borealis* (PelA) has demonstrated significant divergence from well-characterized *Bacillus* pectinases, indicating alkaline pectinase broadly exists in nature. The characteristic of not requiring Ca^2+^ to activate enzyme activity endows it with broader application prospects compared to classical pectinases in food (calcium-free juice clarification to improve quality) and textile (eco-friendly plant fiber degumming) sectors. The strategy of enhancing gene dosage through the construction of tandem expression cassettes has effectively increased the secretory level of PelA. Following the optimization of various parameters, we successfully achieved high-level secretory expression of alkaline pectinase to 7520 U/mL. Notably, future work should validate this process at pilot and large scales to confirm industrial scalability. Overall, this study lays a foundation for PelAs industrial application and provides a reference for enzyme expression optimization. Follow-up pilot/large-scale validation and application exploration will facilitate its industrial bioapplication.

## Figures and Tables

**Table 1:** The copy number of pectate lyase gene *pelA* in the genome of yeast recombinants detected by Quantitative PCR.

	Ct Mean of *pelA* Gene	Copy Number *pelA* Gene in the Reaction	Ct Mean of *gapdh* Gene	Copy Number of *gapdh* Gene in the Reaction	Copy Number of *pelA* in a Genome
AO-2	4.738	5.265 × 10^7^	16.700	4.930 × 10^7^	1.068
AO-4	5.096	5.008 × 10^7^	16.501	5.090 × 10^7^	0.984
2AO-2	3.927	9.930 × 10^7^	16.503	5.010 × 10^7^	1.982
2AO-4	3.922	9.955 × 10^7^	16.495	5.079 × 10^7^	1.960
3AO-3	3.291	1.525 × 10^8^	16.498	5.095 × 10^7^	2.993
3AO-4	3.304	1.513 × 10^8^	16.501	5.058 × 10^7^	2.992

## Data Availability

The original contributions presented in the study are included in the article. Further inquiries can be directed to the corresponding author/s.
